# Highly Dispersion Cu_2_O QDs Decorated Bi_2_WO_6_ S-Scheme Heterojunction for Enhanced Photocatalytic Water Oxidation

**DOI:** 10.3390/nano12142455

**Published:** 2022-07-18

**Authors:** Diyong Tang, Desheng Xu, Zhipeng Luo, Jun Ke, Yuan Zhou, Lizhong Li, Jie Sun

**Affiliations:** 1 Key Laboratory of Resources Conversion and Pollution Control of the State Ethnic Affairs Commission, College of Resources and Environmental Science, South-Central Minzu University, Wuhan 430074, China; 2020120750@mail.scuec.edu.cn (Z.L.); 3027407@mail.scuec.edu.cn (L.L.); jetsun@mail.scuec.edu.cn (J.S.); 2 School of Chemistry and Environmental Engineering, Wuhan Institute of Technology, Wuhan 430073, China; xudesheng123@outlook.com (D.X.); jke@wit.edu.cn (J.K.); zhouyuansun@hotmail.com (Y.Z.)

**Keywords:** photocatalysis, S-scheme heterojunction, water oxidization, quantum dots

## Abstract

Developing suitable photocatalysts for the oxygen evolution reaction (OER) is still a challenging issue for efficient water splitting due to the high requirements to create a significant impact on water splitting reaction kinetics. Herein, *n*-type Bi_2_WO_6_ with flower-like hierarchical structure and *p*-type Cu_2_O quantum dots (QDs) are coupled together to construct an efficient S-scheme heterojunction, which could enhance the migration efficiency of photogenerated charge carriers. The electrochemical properties are investigated to explore the transportation features and donor density of charge carriers in the S-scheme heterojunction system. Meanwhile, the as-prepared S-scheme heterojunction presents improved photocatalytic activity towards water oxidation in comparison with the sole Bi_2_WO_6_ and Cu_2_O QDs systems under simulated solar light irradiation. Moreover, the initial O_2_ evolution rate of the Cu_2_O QDs/Bi_2_WO_6_ heterojunction system is 2.3 and 9.7 fold that of sole Bi_2_WO_6_ and Cu_2_O QDs systems, respectively.

## 1. Introduction

Sunlight provides an abundant renewable energy source to overcome the energy crisis that humans face in the future. Among all the strategies, solar energy conversion from sunlight into chemical energy has shown up as a sustainable and efficient route utilizing semiconductor photocatalysts [1,2]. As we know, water oxidation to dioxygen is a multi-electron transfer reaction in a photocatalytic water splitting process, which is a critical step and involves the difficult breaking of the O–H bond as well as the formation of an O–O bond [3,4]. Continuous efforts have been dedicated to the development of efficient water oxidation catalysts (WOCs), consisting of desirable semiconductor photocatalysts and cocatalysts with proper band structure and electrophilic ability, which could improve the light absorption capability and charge transportation with overall promoted photocatalytic performance for water oxidation [5,6].

Among various semiconductor photocatalysts, ternary metal oxide, *n*-type Bi_2_WO_6_, as one of the simplest members of the Aurivillius family, is comprised of accumulated layers of perovskite-like [WO_4_]^2−^ octahedral sheets and [Bi_2_O_2_]^2+^ sheets [7,8,9]. Density functional theory (DFT) calculations show that the conduction band (CB) of Bi_2_WO_6_ is comprised of W 5d orbitals; the valence band (VB) mainly originates from hybridizing O 2p with Bi 6s orbitals, which not only enables the VB to be highly dispersed, but also facilitates the migration of photogenerated holes for specific oxidation reactions. In addition, the band gap of Bi_2_WO_6_ is about 2.8 eV, and the valence band edge is at +2.95 V vs. NHE (normal hydrogen electrode), which is high enough to trigger the water oxidation reaction for oxygen production. These unique properties reveal that Bi_2_WO_6_ can be utilized as a visible-light-driven photocatalyst for organic synthesis, CO_2_ reduction, and environmental remediation [10,11,12]. Nevertheless, similar to many semiconductors, the poor utilization efficiency of solar energy and high recombination rate of pure Bi_2_WO_6_ give rise to depressed photocatalytic activity and thereby cannot meet the rising demand of commercial applications [13,14,15].

Compared with mono-component photocatalysts, the hybrid heterojunction photocatalysts that hybridize at least two different functional catalysts into one system have attracted increasing attention in recent decades. In particular, the advanced Z- or S-scheme heterojunctions have been extensively investigated and reported; they synchronously realize efficient separation, transportation, and utilization of photoinduced charge with strong redox abilities by means of recombining weak electrons and holes at low potentials between the two semiconductors [16,17]. Therefore, to enhance the photocatalytic efficiency of Bi_2_WO_6_, it is feasible to couple Bi_2_WO_6_ with other cocatalysts for constructing an S-scheme heterojunction system [18,19,20]. For example, Liu et al. constructed Bi_2_WO_6_/CoAl-LDHs (layered double hydroxides) S-scheme heterojunction to obtain enhanced photo-Fenton-like catalytic performance, which profited from the synergistic effect of an internal electric field and S-scheme heterojunction [20]. Recently, quantum dots-modified semiconductor functional materials have received tremendous attention [21,22,23]. The quantum dots (QDs) can significantly increase the photon conversion efficiency by generating multiple excitons from a single photon owing to their unique quantum effect, but also easily match well with the band alignment of the host semiconductor [24]. Taking carbon QDs as an example, Kang et al. utilized carbon QDs to decorate Bi_2_WO_6_ for constructing the desirable band structure conditions induced by compensatory photo-electronic effects, thereby realizing overall water photo-splitting [25]. Moreover, the high specific surface area (SSA) supplies numerous active sites which are favorable for the adsorption of reactants and thus enhancing the observed photocatalytic activity. A major merit of these QDs decorated semiconductors is that more micro-heterojunction and a faster charge transfer process can be sustained due to the intimately contacted nature of the interface and the short charge-carrier transport paths. Among numerous semiconductors, Cu_2_O QDs have shown up as a good candidate for tailoring photo-response and promoting charge carrier migration properties because of the well-aligned overlapped band structures of Bi_2_WO_6_ and Cu_2_O [26]. In fact, Cu_2_O is widely applied as an effective co-catalyst in photocatalytic or electrocatalytic systems for a hydrogen evolution reaction (HER) owing to its high conduction band potential, exhibiting good photocatalytic H_2_ production activity [27,28].

In this study, we successfully decorated Cu_2_O QDs onto the surface of Bi_2_WO_6_ micro-flowers (MFs) with a uniform dispersion to form multiple S-scheme micro-heterojunctions for enhancing the efficiencies of solar light utilization and photogenerated charge migration. The incorporation of Cu_2_O QDs improved the adsorption ability of visible light and effectively facilitated the transportation of photoinduced charge carriers, and thus enhanced the photocatalytic activity for oxygen production under simulated solar light irradiation. This work suggests that the coupling of nanosized *p*-type Cu_2_O QDs and the three-dimensional Bi_2_WO_6_ MFs has a great potential for application in photocatalytic water oxidation.

## 2. Materials and Methods

### 2.1. Synthesis of Flower-Like Hierarchical Bi_2_WO_6_ MFs

In a typical procedure, 1.32 g of Na_2_WO_4_·2H_2_O was dissolved into 40 mL of purified water to form a transparent solution. Meanwhile, 1.96 g of Bi(NO_3_)_3_·5H_2_O was firstly mixed with 80 mL of HNO_3_ (0.3 M). After that, the Na_2_WO_4_ solution was dropped into the Bi(NO_3_)_3_ solution with vigorous magnetic stirring, and a white precipitate was formed quickly. Subsequently, 20 mL of NaOH solution (0.2 M) was added dropwise with stirring for 12 h. Finally, the mixture was transferred to a Teflon-lined autoclave and kept at 160 °C for 8 h. A light-yellow precipitate Bi_2_WO_6_ MFs was centrifuged, washed by purified water and dried in air at 60 °C.

### 2.2. Synthesis of Cu_2_O QDs/Bi_2_WO_6_ Heterojunction

Firstly, 0.025 g of hexadecyl trimethyl ammonium bromide (CTAB) was dissolved into 20 mL of purified water to form transparent solution. Then, 0.1 g of the as-prepared Bi_2_WO_6_ sample was added into the above CTAB solution with stirring for 30 min. Meanwhile, 0.008 g of copper acetate (Cu(Ac)_2_) and 0.016 g of ethylenediaminetetraacetic acid disodium (EDTA-Na) were dissolved into 5 mL of purified water. Subsequently, the Cu solution was mixed with the Bi_2_WO_6_ solution. Then, 10 mL of NaOH solution (0.05 M) was added dropwise into the mixed solution with stirring for 30 min. Afterwards, 10 mL of ascorbic acid (AA) solution (0.33 g) was dropped into the above solution with vigorous stirring for 1 h. The generated Cu_2_O/Bi_2_WO_6_ was washed with absolute ethanol and distilled water several times to remove the surfactant, and dried overnight in a vacuum oven. The final products were named 1.5 wt% Cu_2_O/Bi_2_WO_6_, 3wt% Cu_2_O/Bi_2_WO_6_, and 6 wt% Cu_2_O/Bi_2_WO_6_, where the 1.5, 3 and 6 wt% were the mass ratios of Cu_2_O to Bi_2_WO_6_ in the mixed solution according to the theoretical stoichiometric ratio of added copper and bismuth elements. For comparison, a control sample was prepared without the addition of Bi_2_WO_6_ and labeled as Cu_2_O.

### 2.3. Characterizations

X-ray diffraction (XRD) patterns of the prepared heterojunctions were performed using a Bruker D8 diffractometer (Billerica, MA, USA). The morphology and microstructure of the obtained catalysts were observed using a JSM5510LV (Tokyo, Japan) field emission scanning electron microscopy (SEM) and a JEOL 2100 (Tokyo, Japan) transmission electron microscopy (TEM). Raman spectra were recorded on an ISA dispersive Raman spectroscopy at 514 nm. Fourier transform infrared spectra (FTIR) were determined using a Bruker spectrometer (Billerica, MA, USA) with an ATR correction mode. X-ray photoelectron spectroscopy (XPS) was examined by a Thermo Escalab 250 instrument (Waltham, MA, USA) with Al-Kα radiation to determine the surface chemical species. UV–vis absorption spectra were conducted by a Cary 4000 UV-vis spectrometer (Waltham, MA, USA). Electron paramagnetic resonance (EPR) analyses were carried out using a Bruker EMS-plus instrument (Billerica, MA, USA) to detect the free radicals by using 5,5-dimethyl-1-pyrroline (DMPO) as a spin-trapping agent.

### 2.4. Photoelectrochemical Tests

Photoelectrochemical measurements were conducted using a CHI660E electrochemical workstation (Shanghai, China) with a three-electrode system in 0.05 M Na_2_SO_4_ electrolyte (20 mL, pH = 6.8). A catalyst deposited fluorine-doped tin oxide (FTO) electrode was served as a photoanode, while a Pt wire and a saturated calomel electrode (SCE) were applied as the counter electrode and reference electrode, respectively. For the photoanode preparation, 40 mg of the prepared photocatalysts were added into 2 mL of ethanol with 40 μL Nafion solution (5 wt%) and mixed homogeneously using a vortex oscillator. After that, the resulting mixture was dip-coated onto the prewashed FTO glass to obtain a film electrode with a controlled electrode area of 1 cm^2^. The solar light source (I_0_ = 100 mW cm^−2^) was simulated using a 200 W Xenon lamp coupled with an AM 1.5G filter. Electrochemical impedance spectroscopy (EIS) tests were measured at a scan frequency range of 0.1 to 100 kHz under a voltage amplitude of 10 mV and a potential bias of 0.298 V vs. SCE.

### 2.5. Photocatalytic Activities

The photocatalytic reactions were performed in a Teflon lining reactor under the simulated solar light. 0.05 g of samples were added into 200 mL of the solution with La_2_O_3_ (0.2 g) and AgNO_3_ (0.03 M). Before irradiation, the mixture was stirred for 30 min in the dark and then purged with N_2_ to removal O_2_. The concentration of O_2_ in the reactor was measured by using gas chromatograph (Tet, GC-2030,Tokyo, Japan) with a thermal conductivity at an interval of 30 min.

## 3. Results and Discussion

**Figure 1:**
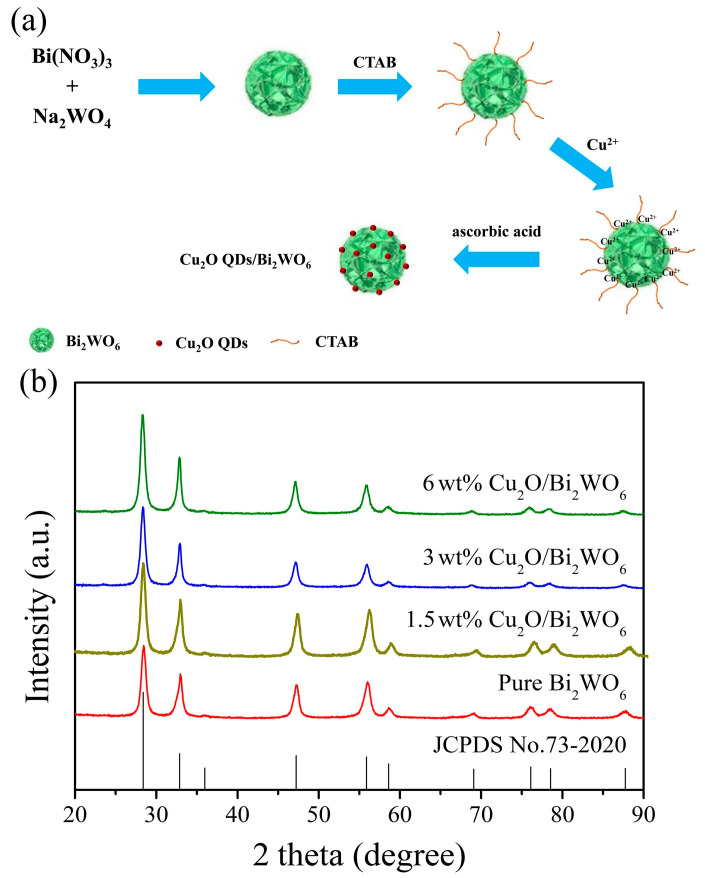
(**a**) Preparation illustrator and (**b**) XRD patterns of Cu_2_O QDs/Bi_2_WO_6_ heterojunction.

Figure 1a displays a possible formation procedure of Cu_2_O QDs/Bi_2_WO_6_ heterojunction through a facile hydrothermal and deposition route. Firstly, when the cationic surfactant CTAB is introduced, the CTAB can be adsorbed and anchored at the surface of Bi_2_WO_6_ MFs. The characteristic flower-like hierarchical Bi_2_WO_6_ with high SSA provides a structural framework for the uniform growth of nanoparticles on the sheets slowly with directed high-density. On the other hand, the EDTA and Cu(Ac)_2_ are mixed with the purified water to form a blue Cu complex. Subsequently, the mixture is added dropwise into the Bi_2_WO_6_/CTAB solution. As a result, the Cu complex is deposited on the surface of flower-like hierarchical Bi_2_WO_6_. With the addition of NaOH, Cu(II) ions from the Cu complex are slowly released to generate Cu(OH)_2_ nanoparticles. As expected, the negatively charged nanoparticles could be attracted and grafted by the positive CTAB to restrain the agglomeration effect. When the weak reductive AA is added, the formed Cu(OH)_2_ nanoparticles can be reduced to Cu_2_O QDs on the surface of Bi_2_WO_6_ MFs, which further maintains the stability of the nanosized Cu_2_O QDs without apparent aggregation. In Figure 1b, the XRD patterns of Bi_2_WO_6_ with different contents of Cu_2_O QDs are present. As displayed, the XRD pattern of the as-prepared Bi_2_WO_6_ is in good agreement with the standard diffraction pattern of orthorhombic Bi_2_WO_6_ (JCPDS No. 73-2020) [29], where the obvious peaks at 28.3°, 32.9°, 47.2°, 55.9°, 58.6°, 69.1°, 76.1°, 78.5°, and 87.7° can be indexed to the (113), (020), (220), (313), (226), (040), (333), (046), and (246) crystal planes, respectively. Moreover, the patterns for Cu_2_O/Bi_2_WO_6_ heterojunctions are similar to those of pure Bi_2_WO_6_, while no characteristic peaks belong to Cu_2_O are observed, which is ascribed to the low loading mass and high dispersion of Cu_2_O QDs in the Bi_2_WO_6_ matrix. 

**Figure 2:**
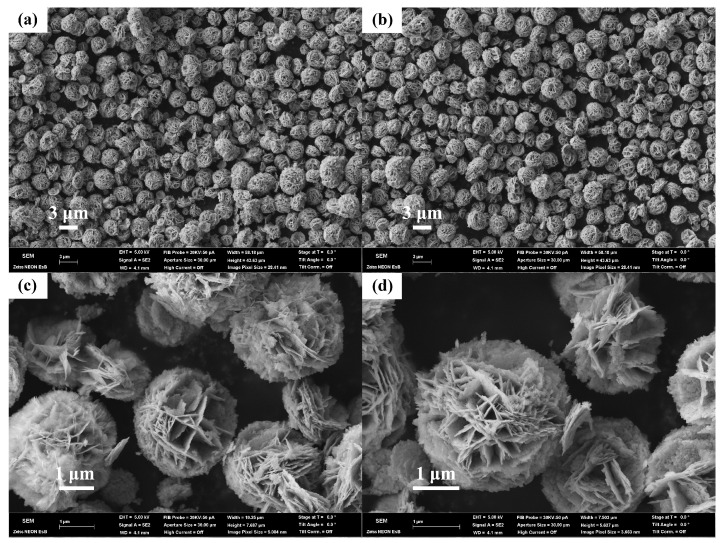
FESEM images of the pure flower-like Bi_2_WO_6_ samples with (**a**,**b**) wide scope and (**c**,**d**) higher resolutions.

SEM images of the bare Bi_2_WO_6_ MFs are displayed in Figure 2a,b, where the uniform flower-like hierarchical Bi_2_WO_6_ with 2–3 μm diameter are observed clearly. It is found that the hierarchical structure of Bi_2_WO_6_ is assembled by ultrathin sheets with 40 nm of thickness, as present in Figure 2c,d, inferring high porosity and huge surface area, which benefits the exposure of more active sites.

**Figure 3:**
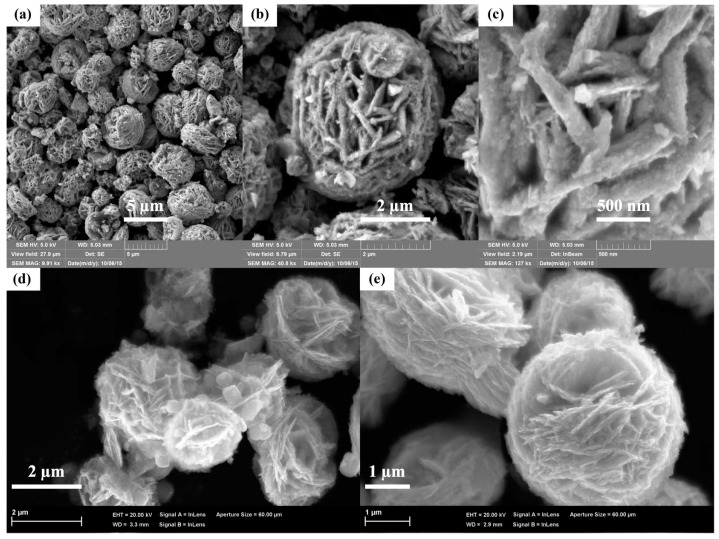
FESEM images of the Cu_2_O QDs/Bi_2_WO_6_ heterojunctions with different Cu amounts: (**a**) 1.5 wt%, (**b**,**c**) 3 wt%, and (**d**,**e**) 6 wt%.

After introducing the Cu_2_O QDs, as shown in Figure 3a,b, it is clearly observed that the size of the Bi_2_WO_6_ hierarchical flowers displays a negligible change, while the nanosheets comprised of the flowers are mechanically exfoliated and the surface of the flower-like hierarchical structure becomes smoother, which is possibly due to the vigorous stirring during the Cu_2_O QDs deposition process. Meanwhile, with the increasing of Cu initial amount, the Cu_2_O nanoparticles are observed and anchored at the surface of the hierarchical Bi_2_WO_6_ MFs. As displayed in Figure 3c, the 3 wt% Cu_2_O QDs are uniformly deposited on the surface of Bi_2_WO_6_ MFs, while once the amount of Cu(II) precursor reaches to 6 wt%, large Cu_2_O nanoparticles are detected in Figure 3d,e, which indicates that the excess Cu(II) precursor is harmful for the dispersion of Cu_2_O QDs and causes the aggregation.

**Figure 4:**
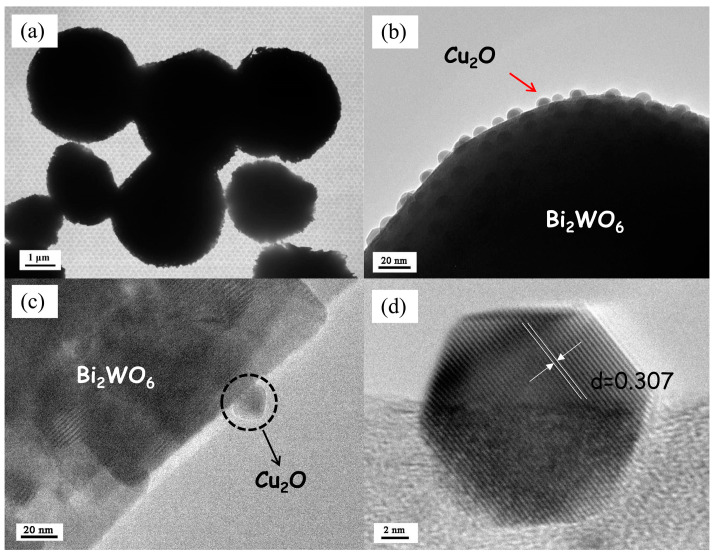
TEM (**a**,**b**) and HRTEM (**c**,**d**) images of the 3 wt% Cu_2_O QDs/Bi_2_WO_6_ sample.

TEM and HRTEM images of the Cu_2_O QDs/Bi_2_WO_6_ heterojunction are presented in Figure 4. The micro-size Bi_2_WO_6_ MFs with 2–3 μm diameter is observed, which is agreement with the results of SEM, as displayed in Figure 4a, where the large thickness of the sample hampers the penetration of electron beams, leading to the black area. In general, quantum dots are defined as semiconductor nanocrystals with particle sizes ranging from 1 to 20 nm, which possess unique electronic properties owing to the apparent quantum confinement effect. It can be clearly observed that the Cu_2_O nanoparticles with ~20 nm of diameter are uniformly dispersed at the surface of Bi_2_WO_6_ MFs in Figure 4b,c. Owing to the smaller size, the Cu_2_O QDs can easily anchor at the surface of micro-sized Bi_2_WO_6_ to form micro-heterojunctions, which shorten the charge-carrier transfer pathways through the intimately contacted interface. The clear lattice fringe of 0.307 nm ascribed to the (110) crystal facet of Cu_2_O is detected in Figure 4d. These results demonstrate the successful construction of heterojunctions between Bi_2_WO_6_ and Cu_2_O.

**Figure 5:**
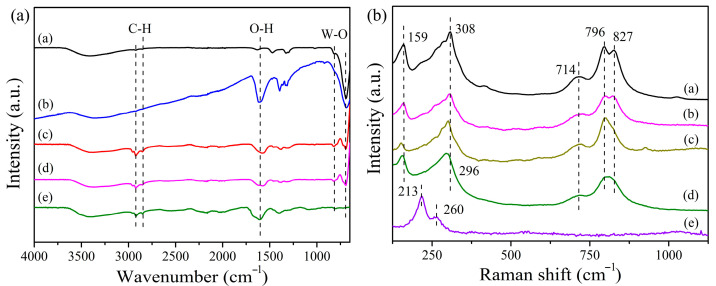
(**a**) FTIR spectra and (**b**) Raman spectra of the as-prepared Cu_2_O/Bi_2_WO_6_ samples: a. Bi_2_WO_6_, b. 1.5 wt% Cu_2_O/Bi_2_WO_6_, c. 3 wt% Cu_2_O/Bi_2_WO_6_, d. 6 wt% Cu_2_O/Bi_2_WO_6_, and e. Cu_2_O QDs.

FTIR spectra of Bi_2_WO_6_ MFs, Cu_2_O, and Cu_2_O/Bi_2_WO_6_ are displayed in Figure 5a. The peaks at 818 and 703 cm^−1^ are attributed to the symmetric and asymmetric vibration of W–O, respectively [30]. The peaks centered at 1599, 2924 and 2845 cm^−1^ are due to the stretching vibration of O–H and C–H, respectively, which could be because of the usage of organic surfactants (CTAB, EDTA) during the synthesis procedure of the heterojunction system [31]. Besides, the characteristic peak of Cu_2_O is not found in the samples of Cu_2_O/Bi_2_WO_6_. To further investigate the composition of samples, Raman spectroscopy of the samples was performed, as shown in the Figure 5b. The characteristic peaks at 796 and 827 cm^−1^ can be ascribed to the antisymmetric and symmetric A_g_ stretch modes of the O–W–O band, respectively [32,33]. The peak at 714 cm^−1^ is associated with the antisymmetric bridging mode of the tungstate chain. In addition, the obvious vibration peak at 308 cm^−1^ is assigned to translational modes involving simultaneous motions of WO_6_^6−^ and Bi^3+^ [34]. For the pure Cu_2_O, the intense peaks at low frequencies of 213 and 260 cm^−1^ originate from the stretching vibration of Cu_2_O, which is consistent with the previous reports [35,36]. In the case of Cu_2_O/Bi_2_WO_6_, the characteristic peak at 308 cm^−1^ shifted to 296 cm^−1^, and the two peaks at 796 and 827 cm^−1^ became a broad peak at 809 cm^−1^ due to the cover of Cu_2_O on the surface of the Bi_2_WO_6_ MFs.

**Figure 6:**
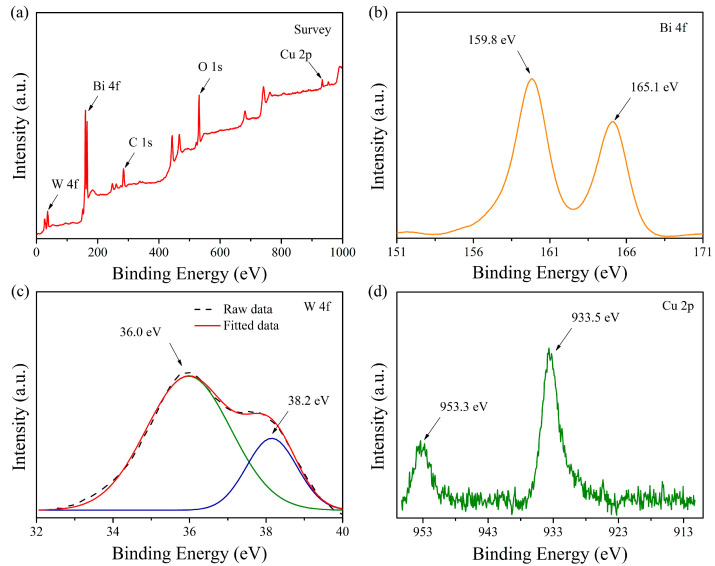
XPS spectra of the 3 wt% Cu_2_O/Bi_2_WO_6_: full spectrum survey (**a**), Bi 4f (**b**), W 4f (**c**), and Cu 2p (**d**).

The XPS spectra were conducted to detect the chemical environment of elements in the catalyst, and all characteristic peaks were calibrated using C 1s (binding energy at 284.6 eV) as a reference. In Figure 6a, elements of W 4f, Bi 4f, O 1s, and Cu 2p were detected in the full survey spectrum of the 3 wt% Cu_2_O/Bi_2_WO_6_, demonstrating the coexistence of these elements in the sample. As presented in Figure 6b, two distinct peaks located at 159.8 and 165.1 eV are assigned to the characteristic peaks of Bi 4f_7/2_ and Bi 4f_5/2_ in the trivalent oxidation state, respectively. In the previous report, the binding energy of Bi 4f_7/2_ in Bi_2_WO_6_ MFs locates in the range of 158 to 159 eV while that for Bi_2_O_3_ appears between 159 and 160 eV. Therefore, the peak located at 159.8 eV could be assigned to Bi^3+^ in Bi_2_WO_6_ MFs [37,38]. In Figure 6c, the high resolution deconvoluted W 4f spectrum reveals two broad peaks at 38.2 and 36.0 eV corresponding to W 4f_5/2_ and W 4f_7/2_, respectively, suggesting the valence state of W element is +6 in the sample of Cu_2_O/Bi_2_WO_6_ heterojunction [39]. Moreover, as seen from Figure 6d, there are two obvious characteristic peaks at 953.3 and 933.5 eV, attributed to Cu 2p_1/2_ and Cu 2p_3/2_, respectively, revealing the feature of Cu^+^ in Cu_2_O [40,41]. In contrast, the CuO state generally has a main characteristic peak locates at a binding energy of higher than 933 eV and characteristic shake-up satellite peaks at around 937–945 eV [42,43,44,45]. The shake-up peaks are often detected at around 9–10 eV higher than the main peaks, which results from the vigorous photoelectrons synchronously interacting with a valence electron and then being excited to a higher binding energy level [46]. However, in Figure 6d, the peak belonging to Cu^2+^ at 933.7 eV with the shake-up peaks at 937–945 eV is not observed, revealing that the copper species in Cu_2_O/Bi_2_WO_6_ hybrids are mainly presented as Cu(I) [47,48,49].

**Figure 7:**
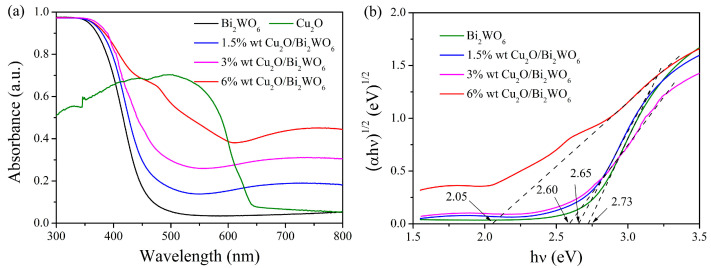
UV–visible absorption curves (**a**) and Tauc’s plots (**b**) of the prepared Bi_2_WO_6_ and different Cu_2_O/Bi_2_WO_6_ heterojunctions.

UV–vis absorption spectra of various heterojunctions and the corresponding band gap energies calculated from the Tauc’s plots by (αh*ν*) = A(h*ν* − E_g_)^1/2^ are presented in Figure 7, which reveals the sunlight response and absorption capability of Cu_2_O, Bi_2_WO_6_ MFs, and various Cu_2_O/Bi_2_WO_6_ hybrids. The absorption edge of Bi_2_WO_6_ MFs is about 460 nm, which suggests that the pure Bi_2_WO_6_ can only absorb UV and near-visible light. However, the absorption spectrum of Cu_2_O sharply rises at the beginning of 650 nm, displaying strong visible light response ability, which makes it a desirable candidate for utilization of solar energy. When depositing Cu_2_O QDs on the surface of Bi_2_WO_6_, the obtained Cu_2_O/Bi_2_WO_6_ hybrid system exhibits improved absorption ability for visible light, as displayed in Figure 7a. The corresponding band gap energies are calculated and displayed in Figure 7b, where the band gap energy of Cu_2_O/Bi_2_WO_6_ hybrids decreases with the introduction of Cu_2_O. Meanwhile, it is observed that the band gap of the 6 wt% Cu_2_O/Bi_2_WO_6_ hybrid is narrowed to 2.05 eV, which is obviously different from those of the 1.5 wt% and 3 wt% Cu_2_O/Bi_2_WO_6_ hybrids. This result suggests that the excess amount of Cu precursor did not result in the formation of Cu_2_O QDs but Cu_2_O microstructures on the surface of Bi_2_WO_6_. It demonstrates that the optimal amount of Cu precursor exists in the formation of QDs-MFs micro-heterojunction structure. On the other word, the excessive Cu precursor leads to the enhancement of sunlight response property.

**Figure 8:**
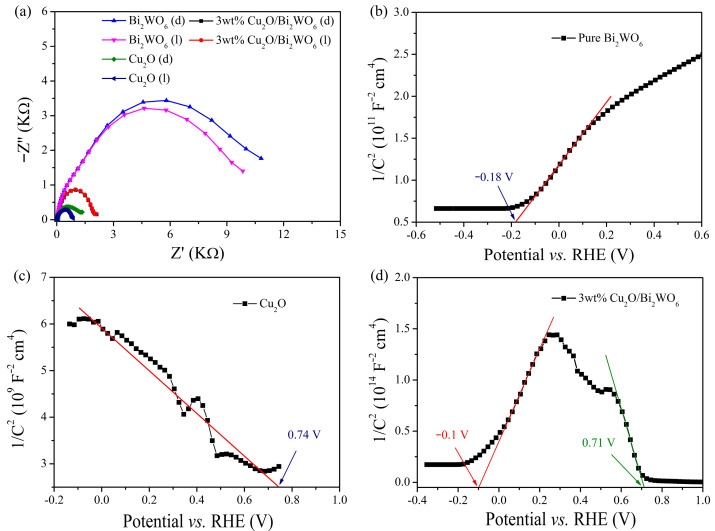
(**a**) Nyquist plots, Mott–Schottky curves of (**b**) Bi_2_WO_6_, (**c**) Cu_2_O, and (**d**) Cu_2_O/Bi_2_WO_6_.

To investigate the transportation behavior and efficiency of photoinduced charge carriers at the heterojunction interface, the photoelectrochemical properties of these samples were investigated. In Figure 8a, electrochemical impedance spectroscopies (EIS) of these samples in the manner of a Nyquist diagram were recorded in the dark and under light irradiation. In general, the radius of each semicircle is correlated to charge-transfer resistance (R_ct_) at the interface of electrode/electrolyte; a smaller semicircle implies a lower R_ct_ value [50,51,52]. As shown in Figure 8a, Cu_2_O exhibits significantly smaller R_ct_ under light irradiation (l) in comparison with being in darkness (d), indicating that the electrical resistance at the electrode/electrolyte interface is decreased due to the production of photoinduced charge carriers. In the case of the flower-like Bi_2_WO_6_ MFs, a larger semicircle is recorded, suggesting that the Bi_2_WO_6_ possesses poor electrochemical performance in charge-transfer process [53,54]. With the formation of the Cu_2_O QDs/Bi_2_WO_6_ heterojunction, the R_ct_ of Bi_2_WO_6_ is intensively reduced, which apparently improves the photoelectrochemical property of Bi_2_WO_6_ and is favorable for the transportation of the photogenerated charge carriers.

To gain deeper insights into the characteristics of the prepared heterojunctions, flat band potential and carrier concentrations are deduced from the Mott–Schottky (M–S) curves [55,56]. The electrode potentials vs. SCE are converted to the reversible hydrogen electrode (RHE) potentials based on the following Nernst equation [57]:

*V_RHE_* = *V_SCE_* + 0.059 × pH + *V*^0^*_SCE_* (1)

where *V_SCE_* is the experimental potential measured against the SCE, *V_RHE_* represents the converted potential vs. RHE, and *V^0^_SCE_* = 0.245 V at 25 °C. The Mott–Schottky (M–S) plots are depicted in Figure 8b–d, in which the flat band potentials at the electrode/electrolyte interface are calculated according to Equation (2) [36]:

1/*C*^2^ = (2/ε_r_ε_0_e*N_d_A*^2^)[(*V* − *V_fb_*) − k*T*/e]c (2)

where *C* is the specific capacity, ε_r_ and ε_0_ are the dielectric constant of the samples and the electric permittivity of vacuum (8.85 × 10^−12^ N^−1^ C^2^ m^−2^), respectively; *N_d_* represents the carrier density of the catalysts, *A* is the efficient area of electrode, *V* and *V_fb_* are the applied working potential and the flat band potential, respectively; k is the Boltzmann constant, *T* donates the absolute temperature, and e is the electron charge (1.602 × 10^−19^ C). In Figure 8b, a positive slope of M–S plot is observed, inferring a *n*-type semiconductor of Bi_2_WO_6_. In contrast, the negative slope of the M-S plot indicates a *p*-type behavior of Cu_2_O in Figure 8c, which is consistent with the previous reports [36,58]. Meanwhile, the flat band potentials of Cu_2_O and Bi_2_WO_6_ are calculated to be 0.74 and −0.18 V vs. RHE at pH = 6.8, respectively. In Figure 8d, an inverted “V-shape” curve is detected in the M–S plot of Cu_2_O*/*Bi_2_WO_6_, which is attributed to a characteristic curve of the *p-n* junction. It demonstrates that two distinct electronic behaviors (*p*- and *n*-type) are exhibited in the Cu_2_O*/*Bi_2_WO_6_ photoelectrode. Moreover, a slight shift of *x* intercept in Cu_2_O*/*Bi_2_WO_6_ occurs, implying the band realignment of Cu_2_O and Bi_2_WO_6_.

**Figure 9:**
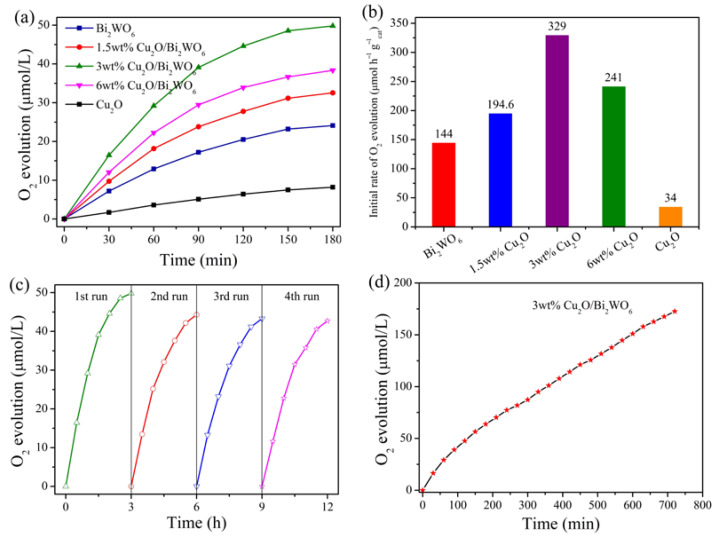
(**a**) Photocatalytic water oxidization performance and (**b**) initial O_2_ evolution rate of these as-synthesized Cu_2_O/Bi_2_WO_6_ heterojunctions. (**c**) Recycling curves and (**d**) stability test of the 3 wt% Cu_2_O QDs/Bi_2_WO_6_ heterojunction.

The photocatalytic water oxidization performances of these prepared samples are presented in Figure 9. As shown in Figure 9a, the Cu_2_O QDs/Bi_2_WO_6_ heterojunctions display significantly enhanced O_2_ evolution activities in comparison with the sole Bi_2_WO_6_ and Cu_2_O QDs under simulated solar light irradiation. The incorporation of Cu_2_O QDs improves the adsorption ability for visible light (Figure 7) as well as electrical conductivity of the prepared Cu_2_O QDs/Bi_2_WO_6_ heterojunctions (Figure 8a), thereby resulting in the enhancement of photocatalytic activity towards water oxidation under solar light irradiation, as the 1.5 wt% Cu_2_O QDs/Bi_2_WO_6_ heterojunction shown in Figure 9a. Meanwhile, the 3 wt% Cu_2_O QDs/Bi_2_WO_6_ heterojunction exhibits the best photocatalytic water oxidation performance, up to 50 μmol/L within 3 h, which is 2.1 and 6.1 times higher than that of pure Bi_2_WO_6_ and Cu_2_O QDs, respectively. Furthermore, the initial O_2_ evolution rate of the 3 wt% Cu_2_O QDs/Bi_2_WO_6_ heterojunction reaches 329 μmol h^−1^ g^−1^, which is 2.3 and 9.7 fold that of sole Bi_2_WO_6_ and Cu_2_O QDs system, respectively (Figure 9b), and is also superior to the reports in the literature (Table 1). However, excessive Cu(II) dosage (6wt%) is harmful for the dispersion of Cu_2_O QDs and causes the aggregation, leading to deteriorated catalytic performance. For the stability of the heterojunction system, as the recycling tests shown in Figure 9c, the photocatalytic performance of the 3 wt% Cu_2_O QDs/Bi_2_WO_6_ hybrid fades to some extent due to the excess deposition of Ag^+^ ions at the surface of heterojunction, but it still maintains good long-term stability and reuse potentiality. As a result, in Figure 9d, the 3 wt% Cu_2_O QDs/Bi_2_WO_6_ hybrid exhibits a sustainable photocatalytic O_2_ production capacity from water splitting.

**Figure 10:**
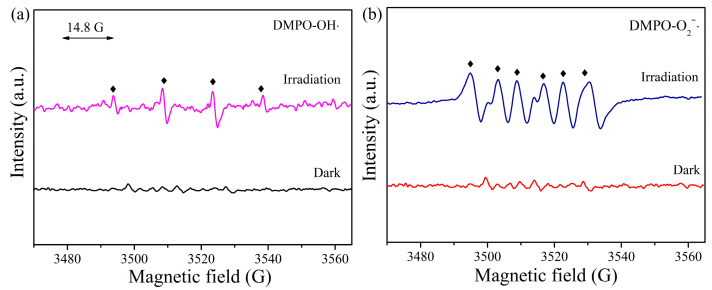
EPR spectra of DMPO-OH∙ (**a**) and DMPO-O_2_∙^−^ (**b**) of the 3 wt% Cu_2_O QDs/Bi_2_WO_6_ heterojunction.

For the 3 wt% Cu_2_O QDs/Bi_2_WO_6_ S-scheme heterojunction, the EPR results are displayed in Figure 10, where the signals attributed to the hydroxyl radicals (·OH) and superoxide radicals (·O_2_^−^) are detected. As shown in Figure 10a, the characteristic four peaks caused by the existence of DMPO–OH∙ adduct are observed, apparently, which demonstrates that water molecular adsorbed on the surface of photocatalyst could efficiently react with the photoinduced holes and form ·OH [69]. On the other hand, in Figure 10b, the characteristic six peaks are clearly found, which is ascribed to the superoxide radical [70]. It is demonstrated that both of ·OH and ·O_2_^−^ can be efficiently produced over the Cu_2_O QDs/Bi_2_WO_6_ hybrids under the solar light irradiation.

**Figure 11:**
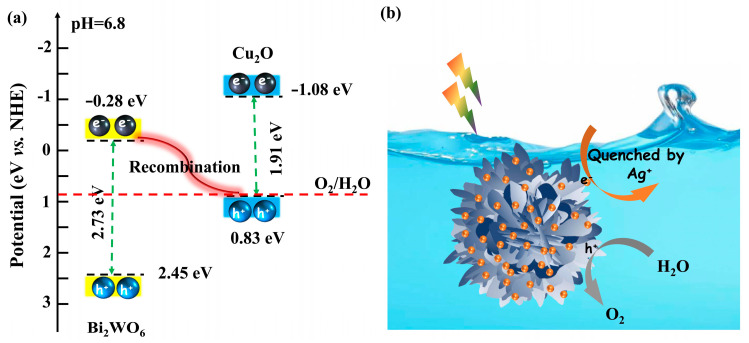
Proposed mechanisms of (**a**) construction of the novel S-scheme band structure and (**b**) photocatalytic water oxidization on the Cu_2_O QDs/Bi_2_WO_6_ heterojunction under simulated sunlight irradiation.

Based on the above results, two types of II or S-scheme heterojunction can be built between Cu_2_O QDs and Bi_2_WO_6_. Once the type II heterojunction is constructed, the trend of photoinduced charge carriers is for photogenerated holes at the VB of Bi_2_WO_6_ to migrate to the VB of Cu_2_O; correspondingly, the photoinduced electrons at the CB of Cu_2_O transfer to the CB of Bi_2_WO_6_. Consequently, photoinduced holes and electrons gather at the CB of Bi_2_WO_5_ and VB of Cu_2_O, respectively. Unfortunately, the VB potential of Cu_2_O is situated at +0.83 eV, which is quite low and makes it hard to guarantee enough oxidative potential to oxidize water and produce gaseous O_2_ [71]. Therefore, it is concluded that the Cu_2_O QDs/Bi_2_WO_6_ hybrids might tend to construct a novel S-scheme band structure, as presented in Figure 11a. The photoinduced electrons at the CB of Bi_2_WO_6_ are likely to quench the holes at the VB of Cu_2_O. Subsequently, the stronger reductive electrons at the CB of Cu_2_O and oxidative holes at the VB of Bi_2_WO_6_ are efficiently retained simultaneously. As described in Figure 11b, the separated photoinduced holes at the VB of Bi_2_WO_6_ react with the adsorbed H_2_O at the surface of hybridized system to generate O_2_, and the retained electrons at the CB of Cu_2_O are quenched by Ag^+^ ions. Therefore, the construction of an S-scheme heterojunction is conducive to inhibiting the recombination efficiency of the photoinduced charge carriers, giving rise to more photogenerated holes taking part in the photocatalytic reactions, thereby enhancing the photocatalytic efficiency towards O_2_ production.

## 4. Conclusions

In summary, we successfully prepared Cu_2_O QDs/Bi_2_WO_6_ heterojunctions by coupling hierarchical Bi_2_WO_6_ MFs with Cu_2_O QDs to construct efficient S-scheme heterojunctions, which could facilitate the migration of photoinduced charge carriers. The electrochemical properties are investigated to explore the transportation performance and donor density of charge carriers in the S-scheme heterojunction system. The results indicate that the synthesized S-scheme heterojunction shows improved photocatalytic activity for water oxidation compared with the sole Bi_2_WO_6_ and Cu_2_O QDs systems under simulated solar light illumination. The initial O_2_ evolution rate of the heterojunction system is 2.3 and 9.7 fold that of sole Bi_2_WO_6_ and Cu_2_O QDs system, respectively. Furthermore, it is evidently demonstrated that both of ·OH and ·O_2_^−^ can be generated efficiently over the Cu_2_O QDs/Bi_2_WO_6_ heterojunction under the simulated solar light illumination.

## Figures and Tables

**Table 1:** Comparison of photocatalytic O_2_ evolution performance between the 3 wt% Cu_2_O/Bi_2_WO_6_ heterojunction and literature reports.

Catalysts	Light Source	O_2_ Evolution Rate in First Hour (μmol h^−1^ g^−1^)	Stability	Ref.
BpCo-COF-1	300 W Xe lamp (λ > 420 nm)	152	4 h	[59]
IrO*_x_*-am@TiO_2_	LED-405 lamp	143.6	4 h	[60]
Mn-BiFeO_3_	300 W Xe lamp (λ > 420 nm)	255	6 h	[61]
BP/BiVO_4_	300 W Xe lamp (λ > 420 nm)	102	3 runs, 9 h	[62]
BiFeO_3_	300 W Xe lamp (λ > 420 nm)	82.2	5 h	[63]
O_v_-BiVO_4_/rGO	300 W Xe lamp (λ > 420 nm)	180	3 runs, 15 h	[64]
Sol-10BP/BiOBr	300 W Xe lamp (λ > 420 nm)	89.5	4 runs, 16 h	[65]
V_Bi_-rich Bi_2_WO_6_	300 W Xe lamp (λ > 420 nm)	100.13	9 h	[66]
KCa_2_Nb_3_O_10_/CoFe-PB	300 W Xe lamp (λ > 420 nm)	89	4 runs, 12 h	[67]
S-BiOCl	200 W Xe lamp (λ > 420 nm)	142	5 runs, 25 h	[68]
3 wt% Cu_2_O/Bi_2_WO_6_	200 W Xe lamp (λ > 420 nm)	329	4 runs, 12 h	This work

## Data Availability

The data presented in this study is available on request from the corresponding author.
